# Organic gelators and hydrogelators

**DOI:** 10.3762/bjoc.6.99

**Published:** 2010-09-21

**Authors:** Jean-Pierre Desvergne

**Affiliations:** 1 Institut des Science Moléculaires, Université Bordeaux 1 - CNRS UMR 5255, 351 cours de la Libération, 33405 Talence Cedex, France

The design and control of molecular self-assembly is of great interest in the development of new molecular architectures with multiple desired functions or properties. In this context, the gelling systems formed by low molecular weight gelators are particularly promising and are the subject of an ever increasing number of studies.

A gel consists of one or more gelling agents and a fluid (organic solvent, water, supercritical liquid) which behaves as a visco-elastic material (soft matter) due to the immobilization of solvent molecules in a three-dimensional network. This network results from the self-assembly of the gelling agent into fibres via non-covalent interactions such as hydrogen bonding, π–π stacking, van der Waals and electrostatic interactions, coordination, and charge transfer. Additional non-covalent interactions lead to physical entanglement of the fibres, which creates a 3D network, the fluid being trapped in the nanoscale interstices. A very large quantity of solvent can be imprisoned in the supramolecular network (in the case of supergelators it is not rare to observe more than 10^4^ molecules of solvent per molecule of gelator in the gel composition), thus creating extraordinary variations of the physical properties of the system. These physical gels are usually thermoreversible (reversible sol-gel transition by heating and cooling) and, depending on the molecular structure of the gelling agent and the fluid which is rigidified, it is possible to form nanoscale superstructures such as nanofibres, nanoribbons, nanosheets, nanoparticles, helical windings, etc., which are of interest for materials and nanoobject conception.

Thus, owing to their non-conventional behaviour, low molecular weight gelators are very attractive for applications in various areas, including supramolecular templates or matrices, transport and release of drugs, art conservation, cosmetics, sensors, optoelectronics, actuators, etc. However, despite numerous efforts to establish a structure-property relationship for the development of low molecular weight gelling agents, prediction of the gelling ability of a compound is not straightforward. A major challenge today is the rational design of small size molecular gelators coupled with an understanding of the mechanism of gelation. It is also important to develop future green gelators for eco-compatible applications.

This thematic series on organogels and hydrogels will address these various points with a particular emphasis on the molecular requirements on the gelling ability, the different approaches for producing molecular gelators, and some techniques used for the characterization and the properties of the gels.

It is my great pleasure to act as guest editor of this Thematic Series, which gathers a wide range of expertise to meet the demands of this interdisciplinary field. I warmly thank all the authors who have enthusiastically accepted to contribute to this series which will give the reader a clear overview of this rapidly developing research field and will identify future growth areas.

Jean-Pierre Desvergne

Talence, July 2010

